# Bladder neck preservation improves time to continence after radical prostatectomy: a systematic review and meta-analysis

**DOI:** 10.18632/oncotarget.11997

**Published:** 2016-09-13

**Authors:** Xueyou Ma, Kun Tang, Chunguang Yang, Guanqing Wu, Nan Xu, Meng Wang, Xing Zeng, Zhiquan Hu, Ranran Song, Bertram Yuh, Zhihua Wang, Zhangqun Ye

**Affiliations:** ^1^ Department of Urology, Tongji Hospital, Tongji Medical College, Huazhong University of Science and Technology, Wuhan, China; ^2^ Department of Maternal and Child Health, School of Public Health, Tongji Medical College, Huazhong University of Science and Technology, Wuhan, China; ^3^ Division of Urologic Oncology, City of Hope National Cancer Center, Duarte, CA, USA

**Keywords:** bladder neck preservation, radical prostatectomy, prostate cancer, urinary incontinence, meta-analysis

## Abstract

Bladder neck preservation (BNP) during radical prostatectomy (RP) may improve postoperative urinary continence, although its overall effectiveness remains controversial. We systematically searched PubMed, Ovid Medline, Embase, CBM and the Cochrane Library to identify studies published before February 2016 that assessed associations between BNP and post-RP urinary continence. Thirteen trials (1130 cases and 1154 controls) assessing BNP versus noBNP (or with bladder neck reconstruction, BNR) were considered suitable for meta-analysis, including two randomized controlled trials (RCT), six prospective and five retrospective studies. Meta-analysis demonstrated that BNP improved early urinary continence rates (6 mo, OR = 1.66; 95% CI, 1.21–2.27; P = 0.001) and long-term urinary continence outcomes (>12 mo, OR = 3.99; 95% CI, 1.94–8.21; P = 0.0002). Patients with BNP also had lower bladder neck stricture frequencies (OR = 0.49; 95% CI, 0.29–0.81; P = 0.006). Anastomotic leak rates, positive surgical margins and biochemical failure rates were comparable between the two groups (P>0.05). There were no differences in baseline characteristics except for a smaller average prostate volume (WMD = −2.24 ml; 95% CI, -4.27 to -0.22; P = 0.03) in BNP patients. Our analyses indicated that BNP during RP improved early recovery and overall long-term (1 year) urinary continence and decreased bladder neck stricture rates without compromising oncologic control.

## INTRODUCTION

Prostate cancer (PCa) is the second most frequently diagnosed cancer among men, and is the fifth leading cause of cancer-related death worldwide [1]. For most men with organ-confined PCa, radical prostatectomy (RP) provides effective oncologic outcomes [2]. The trifecta of optimal outcomes after RP includes preservation of urinary continence, potency and oncologic control [3], and is only achieved in 62–70% of patients [3, 4]. Despite improved surgical techniques, urinary incontinence remains a major postoperative complication, significantly affecting quality of life (Qol) in many men [5, 6]. On average, 16% of men are incontinent 12 months post surgery (using a no-pad definition) [7]. Post-RP incontinence may result in patient preoccupation with leakage avoidance and/or bathroom locations, and feelings of helplessness and embarrassment [8, 9].

To refine the RP technique, Azuma and coworkers [10] suggested a novel surgical approach incorporating “seven key elements of operative skill for the early recovery of urinary continence” (“7 key elements”). Bladder neck preservation (BNP), first introduced in 1992 by Klein [11], has been proposed as a method to accelerate continence recovery, as BNP during RP promotes early return of urinary continence and erectile function [12–16]. However, some clinical trials have suggested little difference in the return of continence with BNP [17–19], and risk of a positive surgical margin (PSM) may be increased [18, 20–22].

The primary objective of this study was to conduct a systematic review and meta-analysis to evaluate the effectiveness of BNP for improving post-RP urinary continence outcomes. The secondary objective was to assess whether or not BNP compromises surgical margin clearance and increases PCa recurrence rate.

## RESULTS

### Characteristics of eligible studies

**Figure 1:**
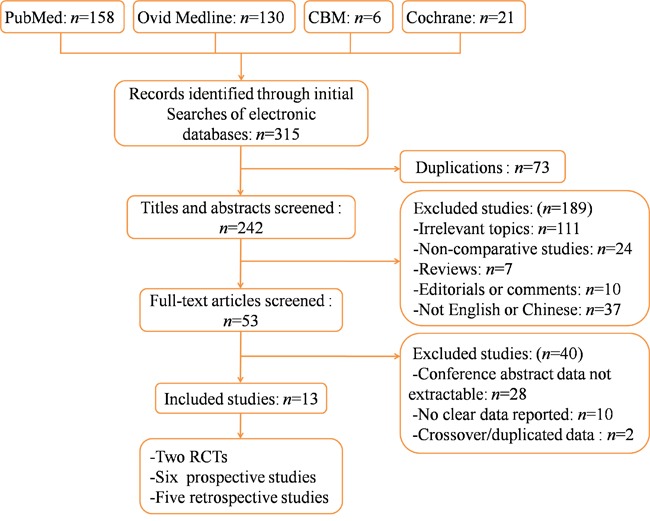
Flow chart illustrating identification and screening of studies RCT, randomized controlled trial.

Thirteen studies with 2284 total participants (1130 cases and 1154 controls) fulfilled the predefined inclusion criteria and were considered suitable for meta-analysis, including two RCTs [18, 23], six prospective [24–29] and five retrospective [19, 30–33] studies (Figure 1).

Study sample sizes ranged between 60 [26] and 619 [25]. Studies were conducted between 1987 and 2012. One prospective study [28] was conducted from August 1987 to August 1998: tennis racquet reconstruction (TRR) was performed from 1987 to 1995 and BNP from 1995 to 1996. Surgical approaches included open, laparoscopic and robot-assisted. Most of the included studies reported BNP versus noBNP (resection or unspecified), and others compared BNP to BNR. One study [19] reported BNP versus BNR and noBNP independently. Baseline continence was poorly reported. Approximately half of the included studies used a no-pad definition for continence; others used a 0–1 pad definition; only one study [28] defined continence as 0–2 pads per day. Timing of continence assessment and reporting ranged from immediately after catheter removal [23, 30, 33] to 24 mo [25].

Demographics, comparative variables of BNP vs. control (noBNP or BNR), continence definitions and follow-up times were extracted individually from each study (Table 1).

**Table 1:** Characteristics of included studies

First author, year	Country	Study interval	Design	Sample size: BNP/noBNP (or BNR)	Matching/comparablea	Surgical approach	Follow-up, mo	Age, year	Timing of outcome, mo	Continence definition
Deliveliotis, 2002 [24]	Greece	1998-2000	prospective	50/51	1,2,4,6	RRP	**NA**	66.1(64-68)/65.2(62-69) c	3,6,9,12	No pad
Freire, 2009 [25]	USA	2005.09-2009.05	prospective	348/271	1,2,3,4,5,6,7,8,9,10	RALP	12.9±9.9/27.1±8.2 b	57.1±6.6/58.9±6.7 b	4,12,24	No pad
Izadpanahi,2014 [26]	Iran	2010.03-2012.03	prospective	30/30(BNR)	1,2,4,6,7,9	RRP	24	60.33±6.96/63.28±7.34 b	2d,18	No pad
Li GH,2013 [30]	China	2009.06-2012.12	retrospective	34/38	1,2,3,5,6,7,8,9,10	EERP	**NA**	63±6/64±7 b	0d,1wk,1,3	0-1 pad
Lou JY,2013 [31]	China	2006.07-2010.05	retrospective	59/86	1,2,3,5,6,8,9	LRP	6	57(42-75)/63.5(46-81) d	1,3,6	0-1 pad
Lowe,1996 [27]	USA	**NA**	prospective	90/98	1,4,5	RRP	35(6-85)/46(6-95) c	61(48-74)/65(47-78) c	1,3,6,12,>12	No pad
Srougi,2001 [18]	Brazil	1998.05-1998.10	RCT	31/38	1,2,4,6	RRP	27(25-30) d	65.2(46-74) d, overall	2d,2,6	0-1 pad
Noh,2003 [28]	USA	1987.08-1998.08	prospective	43/149(BNR)	1,2,6	RRP	≥12	63±7 b, overall	12m	0-2 pad
Nyarangi-Dix,2013 [23]	Germany	2009-2012	RCT	95/104	1,5	RALP, RP	12	63.5±6.5/NA b	0d,1wk,4wk,6wk,3,6,12	0-1 pad
Poon,2000 [19]	USA	1992.09-1997.12	retrospective	101/119 (63, BNR)	1,2,4,7	RRP	17(2-51)/38.5(2-64) d	65(43-76)/64.5(46-76) d	1wk,4wk,3,6,12	0-1 pad
Razi,2009 [32]	Iran	1999-2006	retrospective	51/52(BNR)	1,2,3,6	RRP	32.5(6-84) c	64.8±5.9/65±7.5 b	**NA**	No pad
Stolzenburg,2010 [33]	Greece	2005.06-2008.12	retrospective	150/90	1,2,3,5,7,8,9,10	EERP	≥12	61.3(41-75)/61.6(47-81) d	0d,3,6,12	0-1 pad
You YC,2012 [29]	Korea	2008.01-2010.08	prospective	48/28	1,2,3,4,6,8,9,10	RALP	**NA**	Mean:64.9/65.2	1,3,6,12	0-1 pad

BNP = bladder neck preservation; BNR = bladder neck reconstruction; RP = radical prostatectomy; RRP = radical retropubic prostatectomy; LRP = laparoscopic RP; EERP = endoscopic extraperitoneal RP;

RALP = robot assisted laparoscopic RP; RCT = randomized controlled trail; NA = data not available; PSA = prostate specific antigen; EBL = estimated blood loss

a Matching/comparable variables: 1 = age, 2 = PSA, 3 = prostate volume, 4 = clinical stage, 5 = pathologic stage, 6 =biopsy Gleason score, 7 = pathologic Gleason score, 8 = operation time, 9 = EBL, 10 = catheterization

b Mean±SD

c Mean(range)

d Median(range)

### Demographic and clinical baseline characteristics

**Figure 2:**
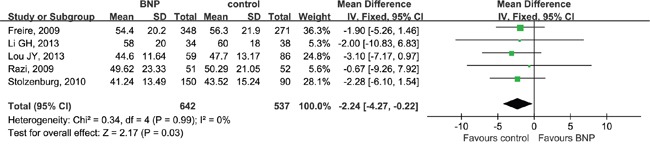
Forest plot of prostate volume CI, confidence interval; IV, inverse variance; BNP, bladder neck preservation; SD, standard deviation. A fixed-effects meta-analysis was conducted (P >0.05, heterogeneity).

There were no significant differences with respect to age, PSA, clinical stage, pathologic stage, biopsy Gleason score or pathologic Gleason score (Table 2). BNP was more commonly performed in smaller-sized prostates (WMD = −2.24 ml; 95% CI, -4.27 to -0.22; P = 0.03). There was no significant prostate volume heterogeneity between studies (Chi^2^ = 0.34, df = 4, I^2^ = 0%; P = 0.99) (Figure 2).

**Table 2:** Analyses of demographic, clinical, pathologic and perioperative characteristics, and oncologic control comparison

Characteristics	No. of studies	No. of patients, BNP/control	OR/WMD (95% CI)	P-Value	Study heterogeneity				Egger test (P-Value)
					Chi^2^	df	P-Value	I^2^	
Age, year	10	1014/898	−1.38 [−2.82, 0.05] a	0.06	82.98	9	**<0.00001**	89%	0.103
PSA, ng/ml	7	722/618	0.14 [−0.08, 0.36] a	0.20	10.86	6	0.09	45%	0.084
Prostate volume, ml	5	642/537	−2.24 [−4.27, −0.22] a	**0.03**	0.34	4	0.99	0%	0.519
**Clinical stage**									
Organ confined ≤ cT2	7	698/636	1.68 [0.61, 4.60]	0.31	1.59	1	0.21	37%	-
Non-organ confined ≥ cT3	7	698/636	0.60 [0.22, 1.63]	0.31	1.59	1	0.21	37%	-
**Pathologic stage**									
Organ confined ≤ pT2	6	776/687	1.02 [0.80, 1.32]	0.85	1.84	5	0.87	0%	0.678
Non-organ confined ≥ pT3	6	776/687	0.98 [0.76, 1.27]	0.88	0.97	5	0.97	0%	0.736
Biopsy Gleason score b	5	233/368	−0.39 [−0.92, 0.13] a	0.14	32.15	4	**<0.00001**	88%	**0.031**
**Biopsy Gleason score**									
≤ 7	2	382/309	1.33 [0.72, 2.44]	0.36	0.73	1	0.39	0%	-
> 7	2	382/309	0.75 [0.41, 1.38]	0.36	0.73	1	0.39	0%	-
**Pathologic Gleason score**									
≤ 7	4	633/516	1.29 [0.90, 1.83]	0.16	1.54	3	0.67	0%	0.862
> 7	4	633/516	0.78 [0.54, 1.12]	0.17	1.61	3	0.66	0%	0.763
Operation time, min	4	591/485	−7.54 [−30.80, 15.73] a	0.53	58.41	3	**<0.00001**	95%	0.130
EBL, ml	5	621/515	1.10 [−40.70, 42.89] a	0.96	24.87	4	**<0.0001**	84%	0.472
Catheterization, day	3	532/399	−0.16 [−0.58, 0.27] a	0.47	0.75	2	0.69	0%	0.663
PSM	13	1130/1155	1.04 [0.81, 1.34]	0.74	9.11	11	0.61	0%	0.634
Biochemical failure	5	276/300	0.78 [0.49, 1.22]	0.27	3.50	4	0.48	0%	0.643

CI = confidence interval; OR = odds ratio; WMD = weighted mean difference; PSM = positive surgical margin

* Statistically significant results are shown in bold.

a Values of WMD

b Biopsy Gleason score (continuous)

### Perioperative variables

We extracted operation times from four studies, estimated blood loss (EBL) from five studies, and length of catheterization from three studies. No differences were observed between BNP and control (noBNP or BNR) with respect to operation time (WMD = −7.54 min; 95% CI, -30.80 to 15.73; P = 0.53), EBL (WMD = 1.10 ml; 95% CI, -40.70 to 42.89; P = 0.96) or length of catheterization (WMD = −0.16 d; 95% CI, -0.58 to 0.27; P = 0.47) (Table 2).

### Relevant complications

**Figure 3:**
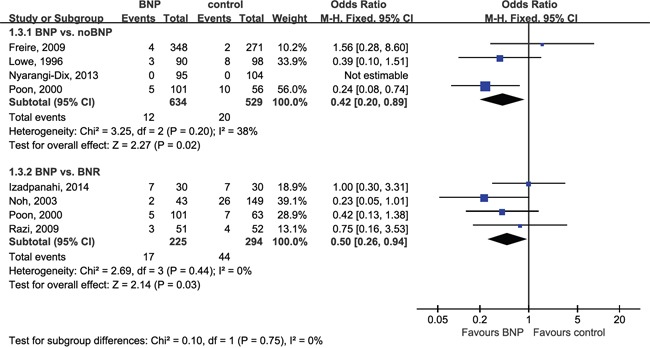
Forest plot of bladder neck stricture CI, confidence interval; M-H, Mantel-Haenszel; BNP, bladder neck preservation; BNR, bladder neck reconstruction. A fixed-effects meta-analysis was conducted (P >0.05, heterogeneity).

Data pooled from seven studies with 1581 total patients with bladder neck stricture associated BNP with lower stricture rates (OR = 0.49; 95% CI, 0.29–0.81; P = 0.006) (Table 3). Subgroup analyses according to surgery type showed differences between BNP and noBNP (OR = 0.42; 95% CI, 0.20–0.89; P = 0.02) or BNR (OR = 0.50; 95% CI, 0.26–0.94; P = 0.03) (Figure 3). In view of one study [19] reported BNP vs. BNR and noBNP independently, the total effect were not calculated directly in Figure 3 but shown in Table 3. There were no differences between BNP and noBNP in urine leak (OR = 1.07; 95% CI, 0.45–2.58; P = 0.88).

**Table 3:** Continence-related outcomes comparison

Outcome of interest	No. of studies	No. of patients, BNP/control	OR(95% CI)	P-Value	Study heterogeneity				Egger test (P-Value)
					Chi^2^	df	P-Value	I^2^	
**Subgroup analyses**									
**BNP vs. noBNP**									
**1. Continence**									
0 d	3	279/232	3.24 [1.61, 6.52]	**0.0010**	1.24	2	0.54	0%	0.074
1 mo	6	385/387	2.45 [1.32, 4.55]	**0.005**	17.57	5	**0.004**	72%	0.288
3 mo	8	585/528	2.04 [1.39, 3.00]	**0.0003**	13.60	7	0.06	49%	0.791
2-4 mo	10	964/837	2.22 [1.42, 3.47]	**0.0004**	33.24	9	**0.0001**	73%	**0.048**
6 mo	8	582/528	1.72 [1.25, 2.37]	**0.0010**	4.25	7	0.75	0%	0.493
12 mo	7	840/675	1.46 [1.06, 2.02]	**0.02**	5.22	6	0.52	0%	0.783
**2. Urine leak**	4	511/459	1.07 [0.45, 2.58]	0.88	2.92	1	0.09	66%	-
**BNP vs. BNR**									
Continence ≥12mo	4	183/279	3.30 [1.26, 8.66]	**0.02**	4.46	3	0.22	33%	**0.025**
**Overall**									
**Continence >12mo**	4	519/451	3.99 [1.94, 8.21]	**0.0002**	5.38	3	0.15	44%	0.218
BNP vs. noBNP	2	438/369	3.96 [1.72, 9.13]	**0.001**	3.62	1	0.06	72%	-
BNP vs. BNR	2	81/82	4.09 [0.98, 17.11]	0.05	1.75	1	0.19	43%	-
**Bladder neck stricture**	7 *	758/823	0.49 [0.29, 0.81]	**0.006**	5.28	5	0.38	5%	0.431
BNP vs. noBNP	4	634/529	0.42 [0.20, 0.89]	**0.02**	3.25	2	0.20	38%	0.112
BNP vs. BNR	4	225/294	0.50 [0.26, 0.94]	**0.03**	2.69	3	0.44	0%	0.686

CI = confidence interval; OR = odds ratio

* overlap of data

** Statistically significant results are shown in bold.

### Urinary continence

**Figure 4:**
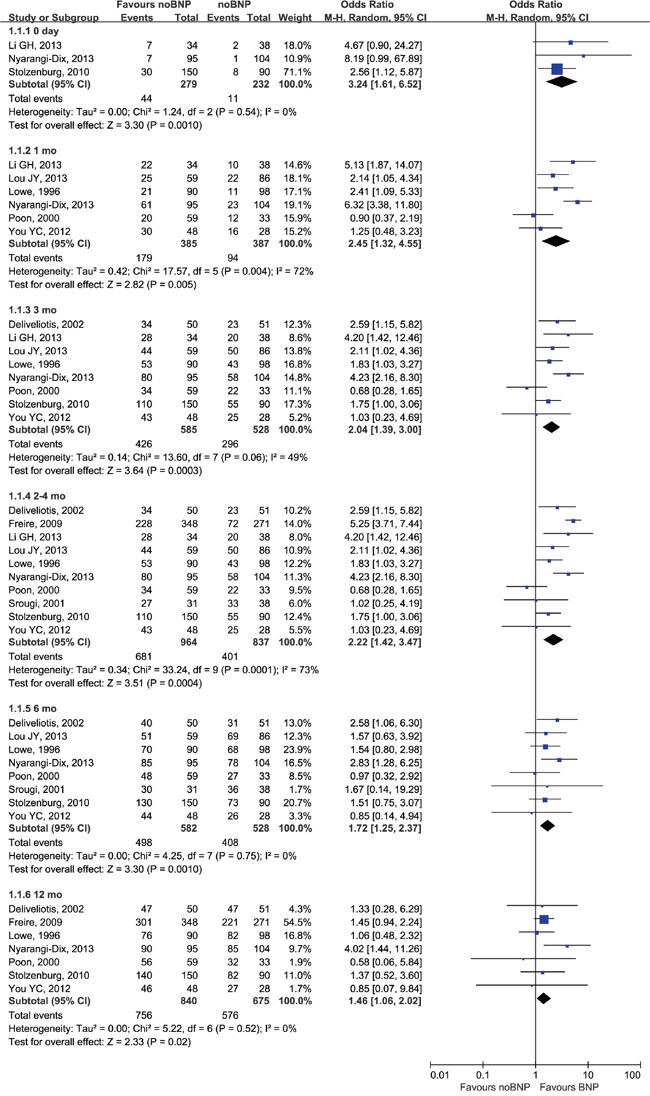
Forest plot of continence rates for BNP vs. noBNP CI, confidence interval; M-H, Mantel-Haenszel; BNP, bladder neck preservation. A random-effects meta-analysis was conducted (P <0.05, heterogeneity).

**Figure 5:**
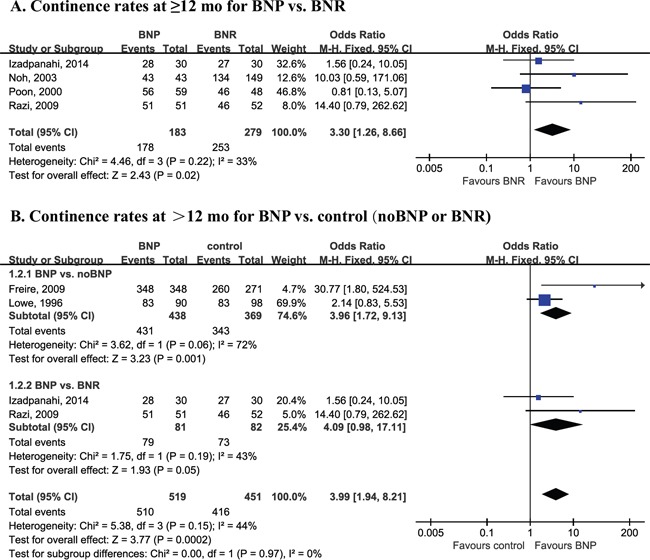
Forest plot of long-term continence outcomes CI, confidence interval; M-H, Mantel-Haenszel; BNP, bladder neck preservation; BNR, bladder neck reconstruction. A fixed-effects meta-analysis was conducted (P >0.05, heterogeneity).

Patients who had BNP surgery had better early and long-term (12 mo) continence outcomes as compared with noBNP (resection or unspecified) surgery (Figure 4). ORs were 3.24 (95% CI, 1.61–6.52; P = 0.0010) at 0 d, 2.45 (1.32–4.55; P = 0.005) at 1 mo, 2.04 (1.39–3.00; P = 0.0003) at 3 mo, 2.22 (1.42–3.47; P = 0.0004) at 2–4 mo, 1.72 (1.25–2.37; P = 0.0010) at 6 mo, and 1.46 (1.06–2.02; P = 0.02) at 12 mo. A difference in continence outcomes with BNP compared to BNR was seen at ≥12mo (OR = 3.30; 95% CI, 1.26–8.66; P=0.02; Figure 5A). Continence differences between BNP and BNR groups at other time points were not subjected to cumulative analysis due to poorly reported data.

Four studies reported long-term results (>12 mo), including 18 and 24 mo. Patients who underwent BNP had better long-term (>12 mo) continence outcomes compared with noBNP or BNR (Table 3). OR was 3.99 (1.94–8.21; P = 0.0002; Figure 5B) at >12 mo. Subgroup analyses according surgery type demonstrated differences between BNP and noBNP (OR = 3.96; 95% CI, 1.72–9.13; P = 0.001) in continence rate at >12 mo; no difference was seen at >12 mo with BNP compared to BNR (OR = 4.09; 95% CI, 0.98–17.11; P = 0.05).

### Oncologic control

Patients with or without BNP had similar PSM outcomes (OR = 1.04; 95% CI, 0.81–1.34; P = 0.74) and biochemical failure rates (OR = 0.78; 95% CI, 0.49–1.22; P = 0.27) (Table 2). Biochemical failure rates were extracted from five studies. However, the definitions of biochemical failure were inconsistent: one study [26] set PSA level limits to 0.4 ng/ml; two [18, 27] set these to 0.3 ng/ml; one [32] set this to 0.2 ng/ml; and one [19] did not mention limits.

### Sensitivity analysis and publication bias

Sensitivity analysis was carried out for prospective studies. There was no change in significance for any outcomes in sensitivity analysis. The funnel plots and Egger test revealed that publication bias existed in only three (Biopsy Gleason score [continuous], continence at 2–4 mo, and continence at ≥12mo) of the 27 comparisons performed in the present analysis (Table 2, 3).

## DISCUSSION

In this systematic review, we analyzed thirteen trials (1130 cases and 1154 controls) to evaluate the efficacy of BNP on urinary continence and its effect on oncologic outcomes. Our meta-analysis included two RCTs, six prospective and five retrospective studies, and demonstrated that BNP surgery improved early recovery and overall long-term (1 year) urinary continence outcomes, decreased bladder neck stricture rates and was effective in eradicating PCa without increasing recurrence rate.

Radical prostatectomy provides effective oncologic control for most men with localized PCa [2]. Despite improved surgical techniques, urinary incontinence remains a chief postoperative complication and affects quality of life in many men [5, 6]. The pathophysiology of post-RP urinary incontinence is not clearly demonstrated. Moreover, the precise anatomy of the bladder neck (BN) and its effect on continence have proven difficult to clarify. The male urethral sphincter complex, composed of an inner smooth muscle lissosphincter and an outer skeletal muscle rhabdosphincter, is essential to continence. In normal physiology, the external urethral sphincter (rhabdosphincter) maintains active continence during stress, whereas the internal urinary sphincter (lissosphincter) provides passive continence at rest [34]. The BN is composed of two different muscles, the ventrolateral and dorsal longitudinal muscles, which are positioned obliquely. In a truly transverse direction of the BN, there is a distinct circular muscle called the musculus sphincter vesicae [35, 36], also named internal urinary sphincter or preprostatic sphincter. In most cases, post-RP incontinence is the result of rhabdosphincter insufficiency [37, 38]. The effect of BNP on early continence outcomes could possibly be explained by preservation of the musculus sphincter vesicae [39], which constitutes an integral part of the male urethral sphincter complex. Additionally, obtaining a BN diameter approximately equal to the diameter of the urethral stump simplifies [40].

Since the first reported anatomic RP, several surgical technique modifications have been proposed to improve early continence recovery and continence outcomes [15, 28, 41, 42]. Some surgeons have attempted to reconstruct the bladder neck by tubularization [43]. Klein [11] first reported the association of BNP with improved early continence. In a multivariate analysis, Sakai et al. [44] reported BNP as the only independent predictor of return to continence at 1 and 3 mo. These results were verified by Gacci et al. [13]. However, other variables such as preoperative pelvic floor muscle exercises and preservation of the neurovascular bundles (NVB) may also influence early continence recovery [45, 46]. Application of preoperative and postoperative continence rehabilitative programs is difficult to control for and was poorly reported in the included studies. Thus, it is unclear if patient groups in each study received these interventions equally.

The effect of BNP on long-term (1 year) continence outcomes has been controversial. Some trials found long-term continence rate benefits from BNP [26, 32, 47], while others did not [24, 28]. This meta-analysis demonstrated improved long-term continence outcomes for patients who had BNP surgery compared with those who had noBNP or BNR (>12 mo, OR = 3.99; P = 0.0002). As comparative data were poorly reported, differences between BNP and BNR groups at other time points were not analyzed except for ≥12 mo. However, the funnel plots and Egger test (Table 3) indicated that publication biases existed in two outcomes: continence at 2–4 mo and ≥12 mo.

Benefits from BNP as compared to controls (noBNP or BNR) with respect to early recovery and overall long-term continence suggest that the bladder neck itself is essential to continence. However, these results are limited by relatively small sample sizes in these analyses.

In addition, type 2 diabetes, baseline continence, different surgical approaches, presenceof other interventions, surgeon experience, surgical technique variations, definition of BNP status, selective outcome reporting and patients lost to follow-up represent risks of bias that could not be controlled for in our analysis. Patients with type 2 diabetes need longer to return to continence than non-diabetics, though this may not affect overall continence [48]. Decreased surrounding tissue damage as a result of careful dissection may also improve urethral preservation and protect supporting continence structures. NVB and urethral length preservation are correlated with improved continence outcomes [46, 49], and highlight the importance of having a detailed knowledge of prostatic and surrounding anatomy in optimal post-RP outcomes. Stolzenburg et al. [50] previously provided an exemplary review of surgical anatomy for RP. Compared with radical retropubic prostatectomy (RRP), patients appeared to benefit from more precise robot-assisted laparoscopic RP (RALP) [51].

Our meta-analysis found no differences between studies regarding patient age, PSA, clinical stage, pathological stage, biopsy Gleason score, pathologic Gleason score, operation time, EBL or length of catheterization. Consequently, our included studies appeared well matched, although variable biopsy Gleason scores (continuous) revealed publication bias.

Selection bias may exist for patients who had BNP, as surgeons may choose patients who are generally fitter to ensure technical ease of preservation. In our meta-analysis, the BNP group had a smaller average prostate size (WMD = −2.24 ml; P = 0.03), which revealed potential bias in selection of patients with low prostate volume. There was no significant heterogeneity between included studies (P = 0.99) that reported prostate size. However, none of the observed differences were seen across all studies. According to a study by Pettus et al. [52], prostate volume is associated with surgical challenges, but not continence outcome after RP.

Heterogeneity existed between studies in terms of operation time (P <0.00001) and EBL (P <0.0001). These could be attributed to differences in surgical approaches, technique, surgeon experience and so on.

Bladder neck strictures are a relatively common, manageable RP complication [26]. PCa patients having BNP surgery may benefit from lower stricture rates (OR = 0.49; P = 0.006). Subgroup analyses demonstrated bladder neck stricture differences in BNP vs. noBNP (OR = 0.42; P = 0.02) and BNP vs. BNR (OR = 0.50; P = 0.03). During reconstruction of the bladder neck (usually a tennis racket method), pressure and tension on the bladder neck tissue due to suture and distortion of normal bladder neck anatomy can promote bladder neck strictures [26]. BNP is also associated with lower rates of ureteral injury [53], which can lead to stricture. Additionally, a larger bladder neck diameter post-RP can result in the need for time-consuming, reconstructive tapering, which may increase susceptibility to anastomotic leak as a result of the longer suture line [25].

With regard to oncologic results, this meta-analysis revealed that patients who underwent BNP had similar outcomes with respect to positive surgical margins (PSM) (OR = 1.04; P = 0.74) and biochemical failure (OR = 0.78; P = 0.27). Some authors argue that BNP surgery may increase the likelihood of PSM [18, 20–22]. However, a randomized controlled trial [23] and other studies [14, 16, 54–56] showed that BNP does not compromise oncologic control. Golabek et al. [16] found that the relatively high incidence of PSM could be due to a large number of extracapsular disease cases.

A major limitation of this study was the small number of well-designed prospective studies. First, there are only two RCTs included in our analysis, along with the six prospective and five retrospective studies. Second, analysis was limited to English- or Chinese-language publications and only published results were included. Third, short follow-up time in some patients, marked heterogeneity for several continuous variables and potential patient selection bias may have influenced the confidence of our results to varying degrees. Additionally, one well-designed study [14] including 1067 patients was not included in the meta-analysis due to absence of discrete data on continence and biochemical recurrence.

To the best of our knowledge, this is the first meta-analysis comparing BNP and noBNP (or BNR) conducted using this type of systematic approach. We applied stringent inclusion criteria to identify studies and compare the two procedures, the Egger test to assess publication bias and sensitivity analysis to minimize the effects of heterogeneity. We provide up-to-date information on the impacts of BNP during RP as compared with traditional techniques. Despite our rigorous systematic approach, because of the inherent limitations of the included studies and the absence of long-term outcomes, further large, prospective, multi-centric, long-term follow-up studies and RCTs should be undertaken to confirm our findings.

## MATERIALS AND METHODS

### Data sources and search strategies

A systematic search of PubMed, Ovid Medline, Embase, CBM and the Cochrane Central Register of Controlled Trials (Cochrane Library) was conducted (February 2016) to identify potentially relevant studies that assessed the association between BNP and post-RP urinary continence. The search was limited to studies published in or after 1992, as BNP surgery was not formally described before that time.

The following terms were searched: [“bladder neck preservation” OR “bladder neck sparing”] AND [“prostatic neoplasms” OR “prostate tumor” OR “prostate cancer” OR “prostatectomy” OR “radical prostatectomy”] AND [“urinary incontinence” OR “continence” OR “postoperative complications”]. The “related articles” or similar function was used to broaden the search, and all abstracts, studies and citations were reviewed.

### Inclusion and exclusion criteria

Articles were further refined through a filtering process based upon the following eligibility criteria: (1) Participants: All subjects were men formally diagnosed with PCa, who underwent radical prostatectomy. (2) Interventions: BNP techniques were defined as bladder neck preservation or bladder neck sparing in radical prostatectomy. (3) Controls: Non-BNP (bladder neck resection or unspecified, noBNP) and active (bladder neck reconstruction, BNR) control conditions were both considered. (4) Outcomes: The primary outcome was urinary continence. (5) Studies: Only controlled trials were considered, including randomized controlled trials (RCT) and non-randomized clinical studies. When multiple publications from the same institution and/or authors with potentially overlapping patient samples were identified, the most recent and/or informative study was included unless the articles were reporting on different outcomes or populations.

Studies were excluded from the meta-analysis if: (1) the inclusion criteria were not met, (2) no outcomes of interest (specified later) were reported or it was impossible to calculate or extrapolate the necessary data from the published results, (3) studies were single-cohort or cross-sectional, (4) the publication language was not English or Chinese.

### Study selection and data extraction

Two reviewers (Xueyou Ma and Kun Tang) separately screened all search results (titles and abstracts). The full text of any potentially relevant publication was retrieved for review and studies were selected based on the criteria previously outlined.

Data were extracted and studies analyzed by two independent reviewers using a standardized data collection form designed by the authors. Any disagreement regarding study selection or analysis was resolved through discussion and consultation with a third reviewer (Zhihua Wang) to reach a consensus. In all cases of missing or incomplete data, the corresponding authors were contacted, but no additional information was provided.

The following data were extracted including: first author, year of publication, country, study interval, study design, number of patients who underwent BNP or noBNP or BNR, surgical approach, baseline characteristics of the study population and outcomes of interest.

### Outcomes of interest

The clinical outcomes that were analyzed and compared between BNP and noBNP or BNR included patient baseline characteristics (age, prostate specific antigen [PSA], prostate volume, clinical stage and pathology results [pathologic stage, biopsy Gleason score, pathologic Gleason score]) and perioperative outcomes (operation time, estimated blood loss [EBL], length of catheterization and relevant complications). Relevant complications included urine leak and bladder neck stricture.

The primary outcome of this review was postoperative urinary continence and the effect of BNP on the timing of urinary continence return after RP. Outcome timing categories (0d, 1, 3, 2–4, 6, 12 and >12 mo) were selected based on all available results. The secondary outcome was evaluation of oncologic control through positive surgical margin (PSM) and biochemical failure.

Some variables were deemed unsuitable for cumulative analysis due to small study numbers. These variables included body mass index (BMI), duration of hospital stay, lymphadenopathy and previous radiotherapy history.

### Statistical analysis

The present meta-analysis was conducted according to the recommendations of the Cochrane Collaboration and the Quality of Reporting of Meta-analyses (QUOROM) guidelines [57]. The weighted mean differences (WMD) and the odds ratios (OR) were used to evaluate continuous and dichotomous variables, respectively. All outcomes were expressed with 95% confidence intervals (CI). For continuous variables (eg, age and length of catheterization), we calculated the difference in mean values and the 95% CI between BNP and control (noBNP or BNR). This method required that studies represent the standard errors of the mean (SEM), the standard deviations (SD) or the CIs. However, some studies did not express any of these parameters, but reported continuous data as medians and ranges. In these cases, we made an approximate transformation using the technique described by Hozo [58]. For dichotomous variables derived from contingency tables (eg, continence rate), ORs and 95% CI were computed. If data were presented as percentages, raw numbers were calculated. An OR significantly <1.0 favored control groups (noBNP and BNR), whereas an OR significantly >1.0 favored BNP groups. All P values are two-tailed with P <0.05 representing statistical significance.

A Mantel-Haenszel fixed-effects (FE) meta-analysis was conducted for dichotomous variable, and Inverse Variance (IV) FE for continuous variable. The quantity of heterogeneity was evaluated using chi-squared and I^2^ statistics with significance set at P <0.05. In cases where higher I^2^ and chi-squared statistic values indicated increasing inconsistency between studies and significant inter-study heterogeneity, a random-effects (RE) model was adopted. Funnel plots and the Egger test of funnel plot symmetry were used to evaluate publication bias.

In forest plots, vertical lines represent the null hypothesis (OR = 1.0), each square represents the point estimate of the OR, and the size of the square represents its relative weighting in the meta-analysis. 95% CIs are depicted by horizontal lines.

Sensitivity analysis was performed by considering studies with RCT or studies clearly of a prospective design. Subgroup analyses according to surgery type (noBNP or BNR) were conducted. Variables were pooled only if studies numbered more than three in the overall meta-analysis.

Statistical analyses were conducted and forest plots generated using Review Manager (RevMan) 5.3 software (Copenhagen: The Nordic Cochrane Centre, The Cochrane Collaboration, 2014). The Egger test was performed using the metabias procedure in STATA12.0 (StataCorp, College Station, TX).
